# Perceived Simultaneity and Temporal Order of Audiovisual Events Following Concussion

**DOI:** 10.3389/fnhum.2018.00139

**Published:** 2018-04-13

**Authors:** Adrienne Wise, Michael Barnett-Cowan

**Affiliations:** Department of Kinesiology, University of Waterloo, Waterloo, ON, Canada

**Keywords:** concussion, time perception, auditory, visual, simultaneity, temporal order

## Abstract

The central nervous system allows for a limited time span referred to as the temporal binding window (TBW) in order to rapidly determine whether multisensory events correspond with the same event. Failure to correctly identify whether multisensory events occur simultaneously and their sequential order can lead to inaccurate representations of the physical world, poor decision-making and dangerous behavior. Damage to the neural systems that coordinate the relative timing of sensory events may explain some of the long-term consequences associated with concussion. The aim of this study was to investigate whether the perception of simultaneity and the discrimination of temporal order of audiovisual stimuli are impaired in those with a history of concussion. Fifty participants (17 with concussion history) were recruited to complete audiovisual simultaneity judgment (SJ) and temporal order judgment (TOJ) tasks. From these tasks, the TBW and point of subjective simultaneity (PSS) were extracted to assess whether the precision and or the accuracy of temporal perception changes with concussion, respectively. Results demonstrated that those with concussion history have a significantly wider TBW (less precise), with no significant change in the PSS (no change in accuracy), particularly for the TOJ task but no significant differences were found between the SJ and TOJ tasks. Importantly, a negative correlation between the time elapsed since last concussion and TBW width in the TOJ task suggests that precision in temporal perception does improve over time. These findings suggest that those with concussion history display an impairment in the perceived timing of sensory events and that monitoring performance in the SJ and TOJ tasks may be a useful additional assessment tool when making decisions about returning to regular work and play following concussion.

## Introduction

A sport related concussion is defined as the rapid onset of a short-lived impairment of neurological function due to a direct or indirect force, which resolves spontaneously (McCrory et al., 2017). While this definition suggests concussion results in short term impairments, the research community is gaining more insight to its long-term consequences and the potential for experiencing prolonged symptoms (McCrory et al., 2017). Long-term consequences include cognitive and motor impairments (Brown et al., 2015; Dalecki et al., 2016), second impact syndrome (Cantu, 1998), chronic traumatic encephalopathy (McKee et al., 2009) and an increased risk of mild cognitive impairment (MCI) and earlier onset of Alzheimer’s disease (AD; Guskiewicz et al., 2005). In sport, accurate and timely assessment of a suspected concussion on the sideline (i.e., at the site or field of play) is critical to prevent athletes from returning to play too soon and risking a subsequent, often more serious, concussion. Currently, sideline assessment tools depend heavily on self-reporting of symptoms (e.g., Sport Concussion Assessment Tool, SCAT; Echemendia et al., 2017) and visually evaluated performance tests (e.g., Vestibular Ocular Motor Screening, VOMS; Mucha et al., 2014). While these tests are valuable for concussion assessment based on symptoms, these tests may be insensitive to subtle deficits associated with mild concussions, leading to these long-term consequences. A deficit that could be associated with concussions that current tests do not account for is impaired perceived timing of sensory events, specifically perceived temporal order and simultaneity. Damage to the neural systems that govern how the central nervous system (CNS) coordinates the relative timing of sensory events may explain problems that concussed individuals continue to experience.

To form a coherent representation of the world, the CNS plays a role in combining multiple sensory modalities accurately together in the temporal domain. However, the CNS faces a multitude of challenges when integrating sensory information to perceive a unified percept and determining the temporal coincidence of events in the environment. Challenges include the differences in the physical propagation of energies in the environment (Spence and Squire, 2003), and the differences in the internal transmission time, processing speed and axonal lengths of each sensory modality (Pöppel et al., 1990). For example, visual stimuli require a longer processing time compared to auditory stimuli (Pöppel et al., 1990). In order for multisensory integration to occur, the stimuli must fall in a specific temporal proximity of one another, known as the temporal binding window (TBW; Meredith et al., 1987; Stein et al., 1998). Thus, the CNS must calculate these temporal latencies between stimuli to determine whether or not information needs to be bound together or remain distinct.

What do we know about the perceived timing of sensory events in the damaged CNS? While research has thoroughly investigated the perceived relative timing of multisensory stimuli in the healthy adult CNS, there is some research that has demonstrated how multisensory information is integrated in the developing and damaged CNS. For example, it has been shown that the width of the TBW shrinks from young childhood and throughout adolescence, settling on a minimal width in young adulthood (Hillock et al., 2011; Hillock-Dunn and Wallace, 2012). It has also been shown that the width of the TBW is extended with damage to the CNS in developmental disorders such as autism (Stevenson et al., 2014) and in patients with Schizophrenia (Capa et al., 2014). Furthermore, an extended TBW has been shown in older adults (Setti et al., 2011; Bedard and Barnett-Cowan, 2016), which can be even more impaired with MCI and AD patients (Wu et al., 2012). Thus, the TBW has the potential for being a sensitive marker for changes in the CNS’s ability to determine the timing of sensory events following global changes to neural function.

The perceived timing of the temporal order and simultaneity of sensory events can be assessed using various psychophysical methods. These methods experimentally manipulate the temporal delay between two sensory modalities—defined as the stimulus onset asynchrony (SOA) between visual and auditory stimuli for example. In these tasks, observers are asked to report their perception of the event, based on the task at hand. A simultaneity judgment (SJ) task asks observers to report whether two stimuli were simultaneous, while a temporal order judgment (TOJ) task asks observers to report which of two stimuli was presented first. From these two tasks, the size of the TBW can be extracted. Additionally, the point of subjective simultaneity (PSS) can be measured—the amount of asynchrony between two sensory stimuli that likely results in the individual perceiving simultaneity (Love et al., 2013).

The purpose of this study is to provide a more detailed understanding of how persons with a history of concussion perceive the temporal order and simultaneity of audiovisual events. Considering the importance of the initial assessment for concussion diagnosis and ongoing clinical assessment for those recovering from concussion, better sideline tools are needed to ensure full functional recovery. Assessing the TBW and PSS in both the TOJ and SJ tasks may provide timely evidence to better inform return to play, work and school decisions. To our knowledge, no studies have considered the relationship between perceived temporal order and simultaneity in those with concussion history. Here, measuring the TBW in those with and without a past history of concussion will be assessed to investigate if and how impaired timing of sensory events is associated in individuals with concussion history. Based on previous evidence of age-related changes in the perceived timing of sensory events (Bedard and Barnett-Cowan, 2016), it is hypothesized that those with concussion history will show a similar TBW width and PSS compared to a healthy control group on the SJ task. For the TOJ task, it is hypothesized that compared to healthy controls, those with concussion history will have an extended TBW width, and require a larger visual lead-time in order to perceive simultaneity.

## Materials and Methods

### Participants

Fifty-one participants with no known auditory or visual deficits were recruited for the study. Participants were recruited through posters located on the University of Waterloo campus. To increase the likelihood of recruiting those with concussion history, athletes from the Waterloo Warriors Varsity program were approached at practices and were invited to participate. Some participants were paid $10/h for their time, while others volunteered to participate with no pay. Following collection, one participant was excluded because of the inability to interpret the data. Participants (*n* = 50; 18–35 years; mean age = 21.9, SD = 2.8) were categorized into one of two groups: a concussion history group or a healthy control group. Participants were eligible to be a part of the concussion group if they had experienced one or more concussion(s) in the past 10 years, and participants were eligible as a healthy control if they had never been diagnosed with a concussion. Categorization into these two groups was based on self-report data. All participants with concussion history reported to be asymptomatic at the time of testing. Seventeen participants were recruited into the concussion history group (*n* Females = 10; Mean age = 21.3, SD = 3.0) and 33 individuals were recruited as healthy controls (*n* Females = 24; Mean age = 21.4, SD = 2.2). This study was carried out in accordance with the recommendations of Canada’s Tri-Council Policy Statement: Ethical Conduct for Research Involving Humans (TCPS2) by the University of Waterloo’s Human Research Ethics Committee with written informed consent from all subjects. All subjects gave written informed consent in accordance with the Declaration of Helsinki. The protocol was approved by the University of Waterloo’s Human Research Ethics Committee.

### Study Design

A between-subjects design was used to assess differences between groups with and without concussion history.

### Experimental Procedure

Participants read and signed the information consent form upon arrival. To confirm their self-reported concussions, participants in the concussion history group were also asked to complete a Concussion Safety Program Injury Report and a SCAT 5 Symptoms Evaluation Form (Echemendia et al., 2017). The Concussion Safety Program Injury Report Form is used by the Athletic Therapy team at University of Waterloo, and consists of questions regarding specific details of an individual’s concussion at the time of injury. The SCAT five Symptom Evaluation form asks individuals to rate the severity of a list of concussion related symptoms on a Likert scale ranging from 0 (no symptoms) to 6 (severe symptoms) out of a possible total severity score of 132. For the purposes of this research, participants were asked to rate their current symptoms relative to before their concussion. For participants who had experienced more than one concussion, these forms were based on their most recent concussion. Additionally, participants in both the healthy control and concussion history group were asked to complete a Clinical Information Form. This form consisted of questions regarding their history of concussion, headaches, migraines, neurological and attention disorders, and whether or not they took medications for any of the above conditions.

Each questionnaire and each task was explained to the participant before being asked to complete it. Participants completed practice trials until it was clear they were familiar with each task, and researchers encouraged participants to ask any questions to clarify confusion before the commencement of each task.

### Experimental Setup

Participants were seated in a dark quiet room with their heads stabilized on a chin rest, which was placed 57 cm away from a MacBook Pro (OS 10.9.5 Maverick, 15 inch (2880 × 1800)) computer. The chin rest was used to ensure their heads were stabilized, and 57 cm was used to assure all participants were viewing the visual stimuli from a consistent distance. VPixx Technologies DataPixx software (version 3.01) was utilized to produce the visual and auditory stimuli accurately in time relative to one another. Visual stimuli were presented on the MacBook Pro monitor as a white circle (0.4°), while auditory stimuli was presented through speakers located adjacent to the computer (Altec Lansing Multimedia computer speaker system, ACS95W). Participants would respond to a trial using either the right arrow button or left arrow button on the MacBook Pro, each button being coded to a different response depending on the task.

### Experimental Tasks

**Figure 1:**
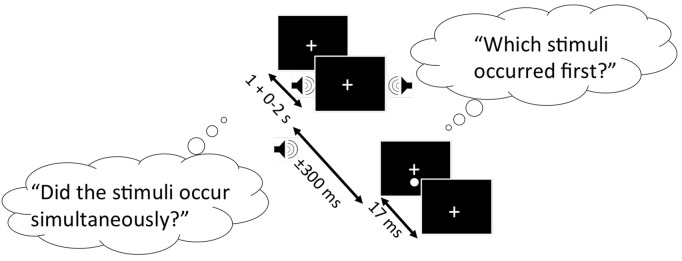
Schematic representation of the simultaneity judgment (SJ) and temporal order judgment (TOJ) task. Black boxes represent the computer screen display, and each bubble represents the questions participants must respond to for the SJ (left bubble) and TOJ (right bubble) task. Arrows indicate the range of time each stimulus is presented. In this specific trial shown, the auditory stimulus (auditory tone) precedes the visual stimulus (white circle).

SJ and TOJ tasks were completed in a randomized order, and are represented in Figure 1 below. Both experimental tasks had an identical design: A trial began when a fixation cross was presented in the center of the computer monitor. Participants were instructed to fixate on this cross throughout the duration of the study. 1.3° below the fixation cross, the visual stimulus was presented for 17 ms against a black background. The visual stimulus was preceded or followed by an auditory stimulus (1850 Hz, 7 ms duration). SOAs were used between the presentation of the two stimuli, including: −300 ms, −200 ms, −150 ms, −100 ms, −50 ms, −25 ms, 0, 25, 50, 100, 150, 200 and 300 ms, where negative values indicate the sound was presented first. Ten trials were completed for each SOA in a randomized order, resulting in 130 experimental trials. All participants were given the opportunity to practice each task until they felt comfortable with completing the experimental trials.

For the SJ task, participants were asked to report whether the visual and auditory stimuli were presented simultaneously (right arrow key) or successively (left arrow key). For the TOJ task, participants were asked to report whether the auditory stimulus (right arrow key) or visual stimulus (left arrow key) was presented first.

### Statistical Analysis

Data analysis was carried out using SigmaPlot version 12.5. As a proxy of the TBW, the just noticeable difference (JND) was measured. The JND indicates the sensitivity of an individual to reliably detect a change of asynchrony between the two stimuli (Harris et al., 2009). To estimate the TBW width and PSS values for each task, SJ and TOJ data were fitted with Gaussian (Equation 1) and sigmoidal (Equation 2) psychometric functions respectively:

(1) y = a⋅ e(−0.5(x−x0 b)2)

where *a* is a scaling factor, *x*ø is the PSS and *b* is the JND (proxy for the TBW).

(2) y = 1001 + ex−x0b%

where *x*ø is the PSS and *b* is the JND (proxy for the TBW).

All participants were included for further analysis. To determine interactions between group and task, independent sample *t*-tests were run to determine significant differences between those with and without concussion history for each parameter for the SJ and TOJ tasks. In cases where normality was violated as assessed by the Shapiro-Wilk test, the Mann-Whitney test was employed. In cases where outliers were identified, the Bayesian independent *t-test* was additionally employed. To assess change of the TBW and PSS as a function of time since last concussion, the Pearson product-moment correlation coefficient was calculated. In cases where TBW or PSS data was not normally distributed, the Spearman’s rank correlation coefficient was employed.

## Results

**Figure 2:**
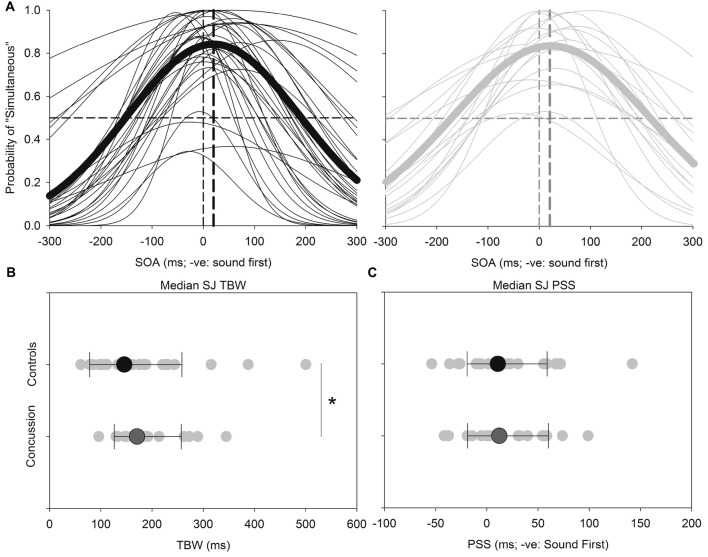
*SJ:* average (*thick lines*) and individual (*thin lines*) SJ Gaussian data fits showing that both healthy adults (*black*) and those with concussion history (*gray*) require the visual stimulus to occur approximately 18–21 ms prior to the auditory stimulus in order to perceive the two stimuli as simultaneous **(A)**. A significantly extended temporal binding window (TBW) was found in those with concussion history, where the difference between medians is shown **(B)**. No differences between medians were found between groups for the point of subjective simultaneity (PSS) **(C)**. *p < 0.05. Error bars are ±1 SD.

Figure 2 shows the results for the SJ task. Both the TBW and PSS values for the SJ task failed normality, and therefore the Mann-Whitney test was employed to determine significant differences between those with and without concussion history. No significant effect was found between those with and without concussion history in terms of the PSS value in the SJ task (*U_(48)_* = 278, *p* = 0.484, Cohen’s *d* = −0.021) For the TBW in the SJ task, a significant between-subject effect was found (*U*_(48)_ = 193, *p =* 0.037, Cohen’s *d* = −0.281).

**Figure 3:**
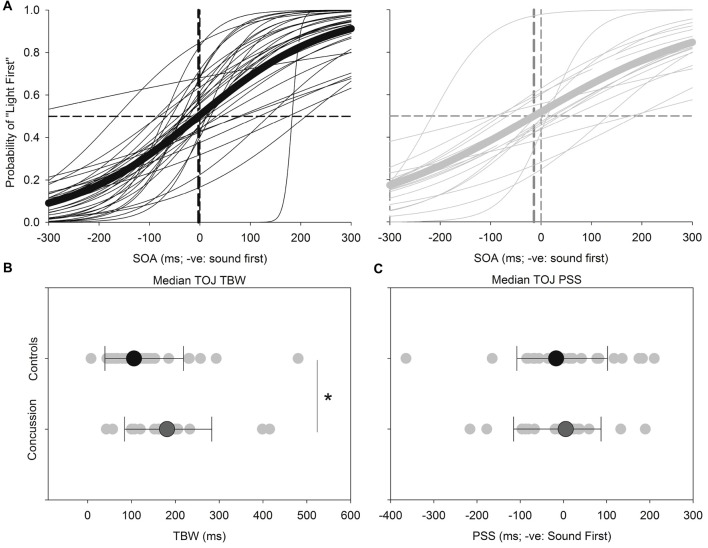
*TOJ:* average (*thick lines*) and individual (*thin lines*) TOJ sigmoidal data fits for healthy adults (*black*) and those with concussion history (*gray*) **(A)**. A significantly extended TBW was found in those with concussion history, where the difference in medians is shown **(B)**. No differences between medians were found between groups for the PSS **(C)**. *p < 0.05. Error bars are ±1 SD.

Figure 3 shows the results for the TOJ task. Both the TBW and PSS values for the TOJ task failed normality, and therefore the Mann-Whitney test was employed to determine significant differences between those with and without concussion history. No significant effect was found between those with and without concussion history in terms of the PSS value in the TOJ task (*U*_(48)_ = 289, *p* = 0.572, Cohen’s *d* = 0.109) For the TBW in the TOJ task, a significant between-subject effect was found (*U*_(48)_ = 163, *p =* 0.008, Cohen’s *d* = −0.588).

Note that statistical outliers were found for the TBW for both SJ and TOJ data and as Bayesian estimation for two groups can handle outliers by describing the data as heavy tailed distributions instead of normal distributions (Kruschke, 2013), we performed Bayesian independent *t*-tests on the TBW values for both tasks between concussed and controls using JASP v0.8.0.1. Here Bayes Factors (BF) provide a numerical value that quantifies how well a hypothesis (H1; concussed TBW significantly different from control TBW) predicts the data relative to a competing null hypothesis (H0; no difference in TBW values across groups), where a BF10 between 0 and 1, indicates support for the H0, and a BF10 greater than 1 indicates support for the H1. Our results show support for the alternative hypothesis (H1) that despite outlier participants being included, the average TBW of the concussed participants was significantly wider than that of control participants for the TOJ (BF10 = 1.378; default Cauchy prior width = 0.707), but not the SJ task (BF10 = 0.422; default Cauchy prior width = 0.707).

**Figure 4:**
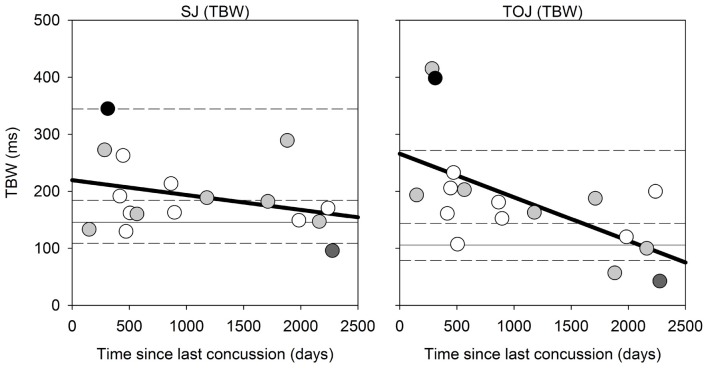
*Correlation Analysis:* correlations for SJ (left) and TOJ (right) between the time since last concussion (days between concussion incident and day of testing) and the TBW width. For instances when only the month and year was given, the first day of the month provided was used. The solid gray horizontal line represents the median TBW for the control group, and the horizontal short dash lines represents the 25%, 75% and 95% percentiles for the control group. Data points are color coded based on number of concussions: white = 1 concussion; light gray = 2; dark gray = 1 individual with 3 concussions; black = 1 individual with ~20 concussions.

Correlational analysis for the SJ and TOJ task is displayed in Figure 4 below, which were conducted using the Spearman’s rank correlation. While the correlation in the SJ task was not found to be significant (Spearman’s rho = −0.316, *p* = 0.216), a significant difference was observed between the time since last concussion and TBW width in the TOJ task (Spearman’s rho = −0.65, *p* = 0.006). For the TOJ task, the less time between the concussion and the time of testing, the wider the TBW. Here two participants were outside the 95th percentile of the control group range for the TOJ TBW. No significant correlations were found for the PSS values in either task. No significant correlations were found between the TBW and PSS as a function of severity of concussion (average severity score: 18.12 out of 132, SD: 19.53).

## Discussion

We investigated changes in the perceived timing of sensory events in those with concussion history, specifically the perceived simultaneity and temporal order of audiovisual stimuli. The results show that compared to a healthy control group, those with concussion history exhibit an extended TBW for audiovisual events and a relationship between the TBW width and time since concussion onset. Evidence in support of this effect was strongest for the TOJ task, though similar trends were found for the SJ task as well. Therefore, the hypothesis is consistent with the findings of this study. To our knowledge, this is the first study that has specifically conducted research that considers the perceived timing of audiovisual events in those with concussion.

While there is currently limited evidence investigating the perceived timing of sensory events in those with concussion history, evidence has demonstrated impaired audio-visual integration with aging, which could help inform potential changes in those with concussion history. For example, while young and older adults have similar TBW widths for the SJ task, older adults have an extended TBW width compared to younger adults in the TOJ task (Bedard and Barnett-Cowan, 2016). Not only does this finding and those from the current study reinforce the theory that there are different underlying perceptual mechanisms for the SJ and TOJ tasks (Love et al., 2013), it also demonstrates the flexibility of the TBW size with changes to the CNS.

What might explain a widened TBW in older adults and those who have had a concussion? A widened TBW in the older adult population found in the TOJ task has been proposed to be associated with the age-related loss of the inhibitory neurotransmitter, GABA (Bedard and Barnett-Cowan, 2016). GABA is an inhibitory neurotransmitter responsible for modulating the excitation of cortical and thalamocortical networks that relay sensory information—such as visual and auditory stimuli (Castro-Alamancos and Connors, 1997). In an aging population, GABA has shown to be reduced. This reduction could lead to less inhibition of irrelevant sensory stimuli and result in behavioral changes such as impaired discrimination of temporal delays (Caspary et al., 2005; Bedard and Barnett-Cowan, 2016). Loss of GABA reduces inhibitory signaling in the CNS, declining the ability to inhibit irrelevant stimuli, therefore leading to their impaired performance of discriminating temporal delays between sensory stimuli (Caspary et al., 2005). Importantly, concussion is also known to alter the relative amounts of glutamate and GABA in the CNS (Guerriero et al., 2015). While these changes in neurotransmitter levels following concussion may not follow a similar pattern to the changes observed in the older adult population, these changes may add noise to signal processing in the CNS. With increased noise in the system, the temporal characteristics of the sensory inputs may not be clearly represented, leading possibly to a wider TBW following concussion.

In addition to the aging population, loss of GABA producing cells has been shown following a brain injury (mild to severe), and the imbalance between GABA and glutamate results in excitotoxicity in the brain (Caspary et al., 2005; Giza and Hovda, 2014). Beyond the initial injury however, chronic changes associated with brain injury are less understood. Using transcranial magnetic stimulation, individuals were shown to exhibit an up regulation in GABA 9 months following concussion, which may be a long-term compensatory mechanism for the excitotoxicity immediately following the injury (De Beaumont et al., 2012b). However, upon looking at individuals 3 years following concussion, no significant differences in neural transmission were found (Tremblay et al., 2014). While these neural transmission changes are different than what is observed in the aging population, as noted above it is possible that the influx of neurotransmitters immediately following concussion and fluctuations throughout recovery may increase the noise in the CNS. With increased noise when processing sensory information, the temporal characteristics of these inputs may not be clear, leading to increased and erroneous integration of sensory stimuli and a widened TBW is observed. However, as GABA neurons interact with other GABA neurons, it is hard to derive any firm conclusion from global changes in GABA/Glutamate levels and their association with the TBW. We therefore suggest that research should continue to understand this potential mechanistic relationship between neural transmissions and TBW width.

Research has demonstrated that those with MCI and AD also exhibit an extended TBW compared to the healthy population when integrating audiovisual information (Wu et al., 2012). Interestingly, one of the long-term consequences of sustaining a concussion is the increased risk of developing MCI and earlier onset of AD. Because MCI and AD show similar sensory integration impairments as those with concussion history, it is possible that this impaired integration that occurs following concussion is a potential factor that increases the individual’s susceptibility to increased MCI risk and earlier onset of AD. The relationship between the TBW and these populations is a potential research avenue that should be further explored.

Not only do the results of this study show a widened TBW in those with concussion, they also demonstrate that the size of the TBW when discriminating the temporal order of stimuli is dependent on the amount of time elapsed since the concussion was sustained. This is yet another case where individuals have returned to play or daily activities, but impairments in neurological function are still present. Research has also shown impaired neurological function following concussion in tasks related to cognitive-motor integration (Brown et al., 2015; Dalecki et al., 2016), episodic memory and response inhibition (De Beaumont et al., 2009) and when completing an audio-visual dual-tasking paradigm (Tapper et al., 2017) all of which cannot be detected by symptom-based assessments. These findings reinforce the need for quantitative metrics to be used by clinicians, in order to make informed judgments regarding patient’s being fully clear of their concussion. The TBW has shown to be a sensitive measure in tracking impaired audiovisual integration over time in those with concussion specifically for the TOJ task. The functional significance of an extended TBW and whether it negatively impacts performance in sport or daily activities is unknown from this research, but could eventually play a role in concussion assessment.

There are limitations to this study that should be addressed. First, with a small and unbalanced sample size, we were unable to examine relationships between the number of concussions and how this affected performance on the audiovisual tasks. Additionally, visual processing deficits are commonly found following concussion, particularly in those with multiple concussions (De Beaumont et al., 2012a). Of 17 participants with concussion two participants reported blurred vision, five participants reported dizziness and nine participants reported sensitivity to light in their SCAT five symptom evaluation form. Therefore a visual test should have been administered to confirm whether these individuals were capable of detecting the visual stimuli, especially given that the duration of the visual stimulus was only 17 ms, as reliably as the rest of the participants who reported no visual changes. However, the two participants with the largest TBWs (Figure 4, right panel with TBWs of ~400 ms) were not the same two participants who reported blurred vision or any visual changes following their concussion(s).

## Conclusion

Here, we have demonstrated an extended TBW in those with concussion history when discriminating the temporal order of audiovisual stimuli compared to the healthy population. Furthermore, we have established a relationship between the size of the TBW with the time elapsed since the concussion when discriminating the temporal order of audiovisual stimuli. These findings suggest that the TBW may be a sensitive marker for tracking the functional recovery of impaired perceived timing of sensory events in those with concussion, and the TOJ task in particular could be a potential candidate test for assessing multisensory integration following concussion. Research should replicate this study with a larger, balanced sample size and controlling for visual deficits, and should explore the relationship between audiovisual integration in concussion and how it may impact sport performance and daily activities.

## Author Contributions

AW and MB-C designed and implemented the study; analyzed the data; wrote the article. AW collected the data.

## Conflict of Interest Statement

The authors declare that the research was conducted in the absence of any commercial or financial relationships that could be construed as a potential conflict of interest.
